# High-Deductible Health Plan Enrollment and Prostate Cancer Screening Rates

**DOI:** 10.1001/jamahealthforum.2025.0180

**Published:** 2025-03-28

**Authors:** Vanessa N. Peña, Daisy Obiora, Chris Kypriotis, Kathryn A. Marchetti, Danielle La Selva, Michael G. Stencel, Bruce L. Jacobs, Benjamin J. Davies

**Affiliations:** 1 Division of Health Services Research, Department of Urology, University of Pittsburgh Medical Center, Pittsburgh, Pennsylvania; 2 Cooper University Healthcare System, Camden, New Jersey; 3 UPMC Center for High-value Health Care, Pittsburgh, Pennsylvania; 4 Charleston Area Medical Center, Charleston, West Virginia

## Abstract

This cross-sectional study assesses the rate of prostate cancer screening in men with high-deductible vs traditional health plans.

## Introduction

The prevalence of high-deductible health plans (HDHPs) has increased over the past 15 years, from 15% in 2007 to 51% in 2023.^1,2^ However, research demonstrates HDHPs indiscriminately lower health care service use, encouraging patients to forgo both needed and preventive care to save costs.^3^ In 2010, the Patient Protection and Affordable Care Act eliminated cost-sharing for preventive services and cancer screenings with a US Preventive Services Task Force (USPSTF) grade A or B. Prostate cancer screening with prostate-specific antigen (PSA) screening is graded C, as it is based on shared patient-physician decision-making, making it subject to out-of-pocket costs. This study assesses prostate cancer screening rates in men with HDHPs vs traditional health plans (THPs).

## Methods

This cross-sectional study included men aged 55 to 69 years enrolled in a large insurance plan in Western Pennsylvania from January 1, 2017, through December 31, 2023. We evaluated the rate of PSA screening with at least 1 PSA test in 24 months of consecutive enrollment in an HDHP or THP. Members with available deductible information and no prior malignant tumors were included. If members had a consecutive enrollment period longer than 24 months, the first 24-month span was used. An HDHP was defined using the annual individual deductible minimum set by the Internal Revenue Service from 2017 to 2023. Coarsened exact matching was used to estimate the likelihood of undergoing PSA screening in an HDHP vs THP after demographic variable adjustment. No patients were eliminated in the matching process. G-computation was used to estimate the marginal effect of plan type on PSA screening probability. A subset of men with a constant deductible was used to perform a semiparametric logistic regression to estimate the association of deductible amount with the probability of PSA screening. This study followed the STROBE guideline. The University of Pittsburgh Institutional Review Board reviewed the study and waived informed consent requirement because the research involved no more than minimal risk to patients and, as a large retrospective medical record review, could not practicably be performed without the waiver. A 2-sided *P* < .05 was considered significant. Analyses were conducted with RStudio, version 2024.04.2 + 764.pro1 (Posit Software).

## Results

A total of 49 905 men were included, with 27 393 enrolled in an HDHP and 22 512 in a THP (Table). In the HDHP group, 49% of men underwent PSA screening compared with 54% in the THP group. Coarsened exact matching analysis found that men in an HDHP were significantly less likely to undergo PSA screening compared with men in a THP (odds ratio, 0.85; 95% CI, 0.79-0.91). G-computation analysis estimated that a 3.6 (95% CI, 2.4-5.6) percentage-point difference in PSA screening probability was attributable to plan type.

**Table.** Characteristics of Patients by Insurance Plan

Characteristic	No. (%) of men aged 55-69 y^a^		*P* value
	Traditional health plan (n = 22 512)	High-deductible health plan (n = 27 393)	
PSA screened	12 069 (54)	13 435 (49)	<.001
Age, mean (SD), y	58.8 (3.0)	58.7 (3.0)	<.001
Race^b^			
American Indian or Alaska Native	23 (<1)	23 (<1)	<.001
Asian	266 (1)	196 (1)	
Black	328 (1)	330 (1)	
Native Hawaiian or Other Pacific Islander	9 (<1)	11 (<1)	
White	14 666 (65)	16 102 (59)	
Other^c^	36 (<1)	28 (<1)	
Missing	7182 (32)	10 703 (39)	
Residence			
Urban	16 455 (73)	20 455 (75)	<.001
Rural	6012 (27)	6864 (25)	
Plan origin			
Exchange	11 876 (53)	10 497 (38)	<.001
Employer sponsored	10 636 (47)	16 896 (62)	
Charlson Comorbidity Index, mean (SD)	0.48 (1.12)	0.37 (0.97)	<.001
Income, mean (SD), $	64 500 (28 200)	66 700 (29 200)	<.001
BA graduate, mean (SD), %	30.2 (18.6)	31.0 (18.8)	<.001
Area Deprivation Index, mean (SD)^d^	60.1 (21.9)	58.5 (22.1)	<.001

Abbreviations: BA, bachelor of arts; PSA, prostate-specific antigen.

^a^ Unless otherwise indicated.

^b^ Self-identified race was included in this study because the financial burden imposed by high-deductible health plans can disproportionately affect people depending on their race. Including these variables allows us to better understand how these plans impact different demographic groups, particularly in terms of access to and use of prostate cancer screening.

^c^ The other race category was available to members who self-identified as a race that was not provided in the list of options.

^d^ Area Deprivation Index is a measure of socioeconomic disadvantage within specific geographic areas based on income, educational level, employment, and housing. The scale is 1 to 100, with higher scores indicating greater deprivation.

A total of 13 655 men had a constant deductible throughout the study period and were used to generate a semiparametric logistic regression model (Figure). In this model, for each $1000 increase in deductible, patients showed a 1% decrease in probability of PSA screening. The greatest change in PSA screening was seen with lower deductibles ($0 to $1386).

**Figure.**
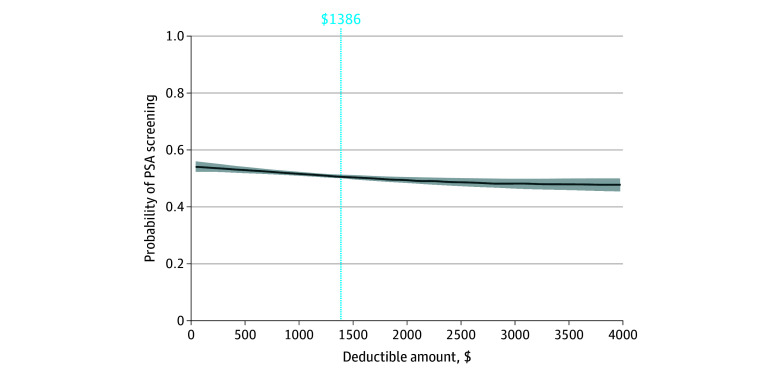
Semiparametric Estimation of Probability of Prostate-Specific Antigen (PSA) Screening as Deductible Amount Increases Shaded areas indicate the 95% CIs. The blue vertical line indicates the mean individual deductible minimum for high-deductible health plans (HDHPs) set by the Internal Revenue Service from 2017 to 2023. Traditional health plan patients with deductibles below this line demonstrated a greater decrease in PSA screening as deductible increased compared with HDHP patients.

## Discussion

In this study, HDHP vs THP enrollment was associated with a lower likelihood of prostate cancer screening in men. These findings suggest that use of PSA screening is sensitive to deductible amount, even among THP patients. Decreased PSA screening rates—witnessed with the COVID-19 pandemic in 2020 and the USPSTF downgrading of PSA screening to grade D in 2012—are associated with increased metastatic prostate cancer incidence.^4,5^ Men younger than 65 years benefit most from prostate cancer screening but are also more likely to have HDHPs.^6^ Thus, the association of HDHPs with screening may be amplified. Limitations include the study’s retrospective nature and inability to assess other forms of cost-sharing, including copayments and coinsurance. Although undergoing PSA screening is a shared decision between patients and physicians, financial motivations are evident. Further research is needed to evaluate the potential association of HDHPs with prostate cancer outcomes.

## Notes

Supplement.
Data Sharing Statement
