# Supplementary material for: High-Deductible Health Plan Enrollment and Prostate Cancer Screening Rates
Source: JAMA Health Forum. 2025 Mar 28;6(3):e250180. doi: 10.1001/jamahealthforum.2025.0180 (PMC11953749; doi:10.1001/jamahealthforum.2025.0180)
Supplement: Supplement. — Data Sharing Statement [file jamahealthforum-e250180-s001.pdf]

Data Sharing Statement

Peña. High-Deductible Health Plan Enrollment and Prostate Cancer Screening Rates. *JAMA Health Forum*. Published March 28, 2025. doi:10.1001/jamahealthforum.2025.0180

Data
Data available: No
